# Evidence of Heating-Dominated Urban NO_*x*_ Emissions

**DOI:** 10.1021/acs.est.4c13276

**Published:** 2025-02-28

**Authors:** Samuel J. Cliff, Will Drysdale, Alastair C. Lewis, Sarah J. Møller, Carole Helfter, Stefan Metzger, Rob Liddard, Eiko Nemitz, Janet F. Barlow, James D. Lee

**Affiliations:** † Wolfson Atmospheric Chemistry Laboratories, University of York, York YO10 5DQ, U.K.; ‡ National Centre for Atmospheric Science, University of York, York YO10 5DQ, U.K.; § UK Centre for Ecology and Hydrology, Bush Estate, Penicuik EH26 0BQ, U.K.; ∥ AtmoFacts LLC., 3570 Larkspur Court, Longmont, Colorado 80503, United States; ⊥ Department of Atmospheric and Oceanic Sciences, University of Wisconsin-Madison, 1225 W Dayton Street, Madison, Wisconsin 53711, United States; # UCL Energy Institute, University College London, London WC1E 6BT, U.K.; ∇ Department of Meteorology, University of Reading, Reading RG6 6BB, U.K.

**Keywords:** air pollution, nitrogen oxides, eddy covariance, natural gas, combustion, decarbonisation, hydrogen

## Abstract

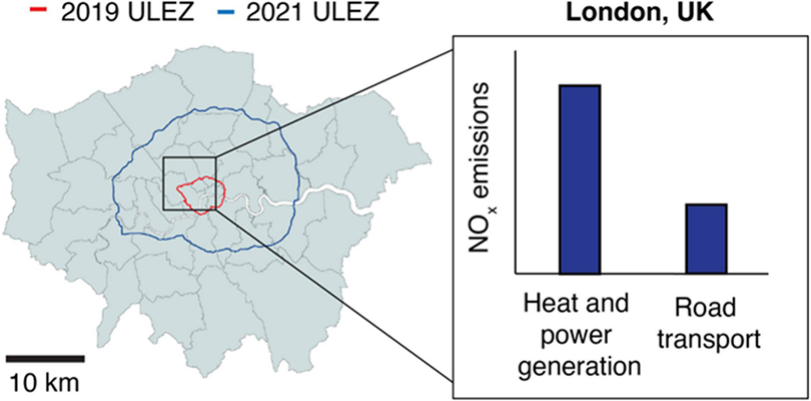

Road transport NO_*x*_ emissions in many high-income countries have steadily reduced due to improved exhaust after-treatment technology. However, ambient concentrations of NO_2_, O_3_ and PM_2.5_ continue to exceed World Health Organization guidelines in many cities globally. The megacity of London has taken an international lead in mobility interventions through the use of low-emission zones. Using long-term air pollution flux measurements made from a communications tower, we show that the largest source of NO_*x*_ emissions in central London has transitioned from road transport to space heating. Observations and supporting consumption/mobility data indicated that natural gas combustion in boilers was responsible for 72 ± 17% of NO_*x*_ emissions in the measurement footprint (average years 2021–2023). Road transport has dominated air quality thinking on NO_2_ for many decades. However, in urban environments that are reliant on natural gas, building heating may now be an effective sector to prioritize for further NO_*x*_ emissions intervention. With system-wide changes in the heat and power sector expected in the coming decades to achieve decarbonisation pledges, we project that very low urban emissions of NO_*x*_ are achievable. The trajectory will, however, depend on choices made around urban buildings and their associated infrastructure and whether low-carbon fuel combustion or electrification pathways are chosen. We estimate a damage cost penalty of up to £600 M in the U.K. should hydrogen combustion replace natural gas for heating rather than technologies such as heat pumps.

## Abstract 2

Evidence that natural gas combustion for heating is of growing importance to urban NO_*x*_ emissions is presented with implications for decarbonisation strategies and future air quality.

## 1 Introduction

Nitrogen oxides (NO_*x*_) play a multifaceted role in the environmental damage of air pollution, contributing to concentrations of criteria air pollutants both directly in nitrogen dioxide (NO_2_), and indirectly via the formation of ozone (O_3_) and particulate matter (PM_2.5_).^1^ In the urban environment, NO_*x*_ are formed via high-temperature combustion. The majority of the production occurs in what is commonly referred to as thermal NO_*x*_ when molecular nitrogen in the air is oxidized via the Zel’dovich mechanism.^2,3^ The rate of NO_*x*_ formation is strongly influenced by temperature and the air-fuel ratio giving different combustion sources different NO_*x*_ emission rates. Historically, emissions in developed urban areas have been dominated by the road transport sector.^4^ This is due to the high fraction of diesel vehicles in European countries and the reliance on the automobile as a method of transport in the US.^5^ The introduction of emissions control technologies like selective catalytic reduction, phased increases in emissions standard stringency, the implementation of traffic pollution/congestion charging zones and fleet electrification has mitigated a large fraction of the emissions.^6−8^ Taking central London, U.K. as an example, a 73% drop in road transport NO_x_ emissions is estimated comparing 2016 to 2025 emissions inventories.^9^ However, the diversity of volatile organic compound sources and the role of biogenic emissions, especially in a warming climate, point toward NO_*x*_ control for future O_3_ regulation in developed cities.^10^ With additional emerging evidence of a supralinear relationship between exposure and health, the continued mitigation of NO_*x*_ is important.^11^

Emissions mitigation in the transport sector has increased the relative importance of other emissions sources, which are often understudied by nature of being less important in the past. The other major source of NO_*x*_ emissions in central London is natural gas combustion in appliances for heating of both water and room space. Typically, the heating sector is broken down into the domestic and nondomestic sectors. Domestic boilers operate in residential homes and are small (typically <50 kW). Nondomestic boilers provide heat to industrial or commercial premises and are much larger (up to 50 MW). In the past, it was generally assumed that the larger the boiler, the higher the operating temperature, and thus the greater the NO_*x*_ emissions. However, “ultralow” NO_*x*_ burner technology for larger gas combustion appliances has been applied for at least a decade.^12^

London is an ideal place to study the importance of heating NO_*x*_ emissions due to (a) the high population density which lends itself to high heating activity, (b) the U.K.’s reliance on natural gas for heating, and (c) the proactive approach the city has taken in reducing emissions from the road transport sector. London has had a congestion charging zone in place since 2003 and more recently implemented, then twice expanded, an ultralow emissions zone (ULEZ). In fact, recent emissions inventory estimates project that heating will be the largest source of NO_*x*_ in Greater London in 2025.^13^

Presented here is a multiyear eddy covariance data set from a tall tower in central London. We outline evidence that the road transport sector now contributes less than natural gas combustion for hot water and space heating to total NO_*x*_ emissions. We highlight that while decarbonisation via electrification to achieve climate change goals will help trend local emissions toward zero, certain strategies for heating infrastructure systems do not necessarily comitigate NO_*x*_ (e.g., low-carbon fuel combustion). As such, we discuss the role of hydrogen/biomass combustion vs electrification for space heating in the context of U.K. air quality ambitions.

## 2 Materials and Methods

### 2.1 Measurement Site and Instrumentation

**Figure 1:**
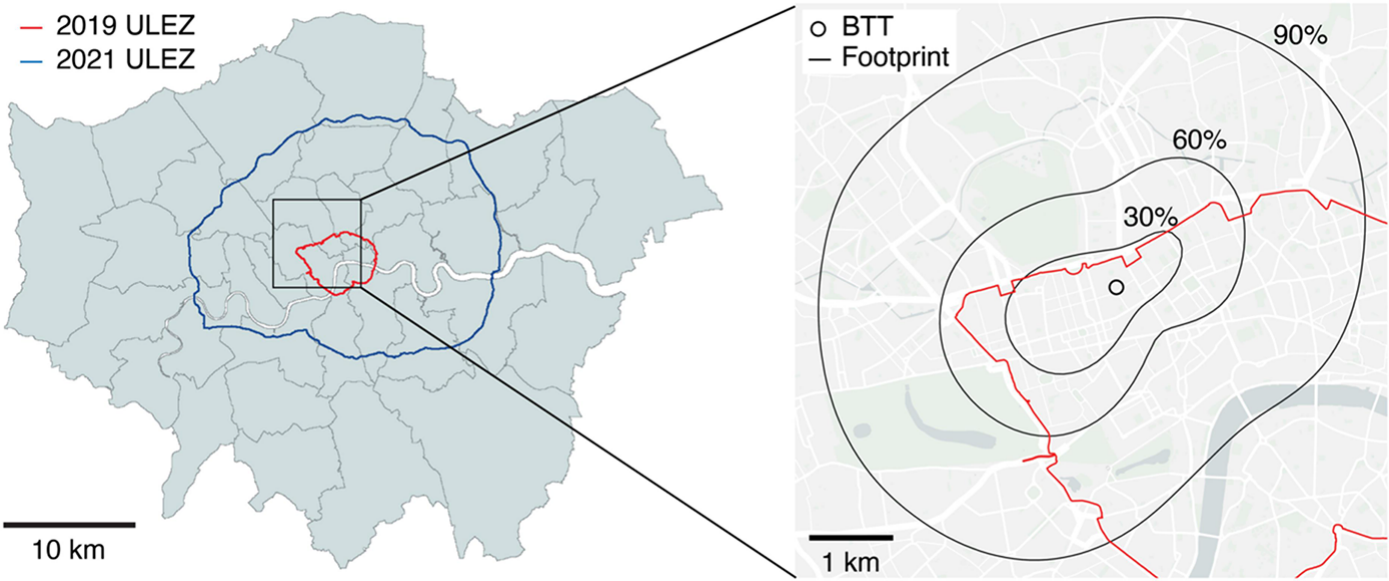
Average measurement footprint for the BT Tower (BTT) displayed as a series of contours, each of which represents a percentage of the contribution. Footprint is shown relative to the Greater London area and the locations of the ultralow emissions zone (ULEZ) boundaries. Map data courtesy of OpenStreetMap contributors, distributed under the Open Data Commons Open Database License v1.0.

Instruments for measuring fluxes of urban air pollutants and greenhouse gases are situated in a small lab atop the British Telecommunications Tower (BT Tower) located in central London, U.K. (51° 31′ 17.4″ N, 0° 8′ 20.04” W, see Figure 1). The measurement height is 190 m above street level, with a mean building height of 8.8 ± 3.0 m in the 10 km radius surrounding the tower.^14^ The gas inlet, ultrasonic anemometer and weather station are mounted on a mast that extends 3 m above the top of the tower. Air is pumped down a 45 m Teflon tube (3/8″ OD) in a turbulent flow of ∼20 L min^–1^ to the gas instruments, which are situated in a small air-conditioned room inside the tower on the 35th floor. The instrumentation used is as follows, with further details referenced herein:

1. Nitrogen oxides (NO_*x*_ = NO + NO_2_): A dual-channel chemiluminescence analyzer (Air Quality Design Inc., Boulder Colorado, USA; 5 Hz; LOD 170 ppt; uncertainty 5%).^8,15^
2. Carbon dioxide (CO_2_): A cavity ringdown spectrometer (Model 1301-f, Picarro Inc., Santa Clara, California, USA; 1 Hz).^8,28^
3. Meteorology (wind speed, direction, temperature, pressure, relative humidity): A sonic anemometer (Gill R3-50, Gill Instruments, Lymington, U.K.; 20 Hz) and a weather station (WXT520, Vaisala Corp. Helsinki, Finland; 1 Hz).^17^

### 2.2 Flux Calculations

The BT Tower site has received extensive characterization over the past 20 years for application to urban flux measurements. This includes applicability to similarity theory and building-induced flow distortion around the meteorological sensor,^14,18^ in addition to numerous campaigns of greenhouse gases (CO_2_, CH_4_)^16,19^ and air pollutant (NO_*x*_ and VOC) flux measurements.^8,15,20,21^ Fluxes were calculated with wavelet-based signal processing using the eddy4R ecosystem within Docker as previously done in tower-based and airborne studies for NO_*x*_ and CO_2_.^22−26^ This facilitates the calculation of high time resolution fluxes with somewhat relaxed assumptions for stationarity. Although we note that the continuity equation underlying eddy covariance simplifications formally still requires stationarity and homogeneity.

Flux calculation via continuous wavelet transformation (CWT) has been described in detail previously in the literature.^27^ Briefly, for simultaneously recorded time series of instantaneous vertical wind *w′*(*t*) and scalar variable *x*′(*t*), the covariance for a given averaging interval is calculated as
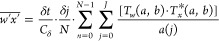
1

The Morlet wavelet was chosen for time series decomposition as appropriate for atmospheric turbulence applications.^27^ Time domain scales were increased linearly at an increment of δ*t* = 0.2 s, and frequency domain scales were discretized using an exponential scale of fractional powers of two.^28^

CWT was performed across 24-h data files with a wavelet maximum scale of 1 h. Fluxes were averaged to 1 min resolution using a 5 min rolling averaging window. No cone of influence filter was applied as previously discussed in the literature;^22^ although performing CWT across 24-hly data files places the greater uncertainty at night when minimal flux is measured (see Figures S1 and S2). All parameters used in the CWT processing are summarized in Table S1. An excellent agreement between traditional eddy covariance and CWT was observed (see Figure S3).

Concentration data was aligned with meteorological data by maximization of the cross-covariance between the two for each hour of measurement data, as outlined in previous studies.^29,30^ Median lag times are approximately 7 s for NO_*x*_ and 20 s for CO_2_. In addition, fluxes were corrected for both high-frequency loss and vertical flux divergence. High-frequency loss resulting from the long sampling line, closed-path instrumentation and insufficient instrument response time was corrected by matching normalized cospectra of wNO, wNO_2_ and wCO_2_ to those of wT (see example spectra in Figures S4 and S5). Corrections were of the order of 2% for NO, 6% for NO_2_ and 16% for CO_2_. Vertical flux divergence resulting from nonuniform turbulence properties in the boundary layer was accounted for using the correction presented in Drysdale et al.^15^ This assumes linear divergence of the vertical flux as a function of effective measurement height and effective entrainment height. Here, hourly ERA5 modeled boundary layer height was used in addition to the measurement height of 190 m to produce an hourly correction.^40,41^ This resulted in an average correction of 30% per data point, although this was weighted to the nighttime data points when fluxes and boundary layer height were lowest. Fluxes were filtered using the eddy4R QAQC criteria such that sufficient turbulence was developed (u* > 0.2), stationarity assumptions were valid, and the measurement height was less than the height of the entrainment layer. Other potential uncertainties, particularly with reactive species such as NO_*x*_, arise from chemical loss during transport to the measurement height. Previously conducted tracer experiments and calculations for the BT Tower site have estimated this as a typical 2% loss rate, increasing up to a maximum of 11% during stable atmospheric conditions.^8,32^ No correction for chemical loss is applied here.

Three years of continuous NO_*x*_ flux data (in addition to two shorter term campaigns)^15,20^ have now been collected at the BT Tower site. The first year of flux data (Sept. 2020–Sept. 2021) was heavily influenced by COVID-19 restrictions and has already been discussed in the literature.^8^ Presented here are the two years of data (July 2021–July 2023) measured after the date on which all restrictions in the U.K. were lifted.

### 2.3 Footprint Modeling

A parametrized version of the backward Lagrangian stochastic particle dispersion model implemented in eddy4R was used to estimate the footprint for each hourly flux measurement at the BT Tower. The model is described by Kljun et al.^33^ and has been parametrized for a range of meteorological conditions and receptor heights. The original model aims to produce a cross-wind integrated footprint function as a function of its along-wind distance, which has now been further extended into two dimensions using a Gaussian distribution driven by the standard deviation in the crosswind component.^34,35^ Meteorology statistics from the eddy covariance calculations are used in combination with modeled boundary layer height from ERA5,^31^ and a surface roughness length of 1.1 m to produce a weighted matrix of 100 m × 100 m grid cells.^36^ Each output weighted matrix was then scaled and aligned to the World Geodetic coordinate reference system. The average footprint for the measurement period is presented in Figure 1.

To obtain a distribution representative of the area that is sampled, all of the spatial data presented in the following sections were weighted by their location within the footprint grid cells. This ensured that geographic areas sampled a greater proportion of the time were appropriately accounted for. It should be noted that ERA5 boundary layer height data used for footprint calculation has a degree of uncertainty and has been shown to underestimate that actually present in the urban environment.^37^ Future boundary layer height measurements are planned in a location within the BT Tower footprint, but in the absence of measurements during this period, ERA5 is used as the best estimate. A sensitivity study of the footprint modeling to boundary layer height and roughness length is provided in the SI.

### 2.4 Source Apportionment

In central London, NO_*x*_ and CO_2_ share road transport and fossil fuel combustion for space heating as their two major sources. The London Atmospheric Emissions Inventory (LAEI) estimates that these two sectors make up >91 and >95% of NO_*x*_ and CO_2_ emissions in our measurement footprint, respectively.^9^ Due to the nature of the combustion processes that fuel these sectors the emitted NO_*x*_/CO_2_ ratio is distinct for each. The measured flux ratio at any given time corresponds to the combination of the ratios of each given sector, and their relative contributions.^38^ Therefore, we use measured NO_*x*_/CO_2_ emission ratios to quantify the contribution of heating and traffic to total NO_*x*_ emissions in central London.

#### 2.4.1 Sector NO_*x*_/CO_2_ Emission Ratios

We estimate temporally varying, London specific NO_*x*_/CO_2_ emission ratios for each of the sectors by adapting those presented in the LAEI. Emissions of CO_2_ typically have a low uncertainty due to generally accurate metering and subsequent national greenhouse gas emissions reporting. This has resulted in well-established emission factors from combustion applications and fuel activity statistics.^39^ Emissions inventory estimates have been shown to agree well with flux measurements in London previously,^19^ and later in this study, and are taken at face value. On the other hand, NO_*x*_ emissions generally have a higher uncertainty due to the variable role of different emissions control technologies. This level of uncertainty varies for different sectors.

Traffic NO_*x*_ emissions have received substantial attention in recent years due to previous inventory inaccuracies arising from the underrepresentation of diesel vehicle emissions under real-world driving conditions. Extensive real-world remote sensing measurements have been conducted which help verify and improve the emissions inventories.^40,41^ For London specifically, the Breathe London campaign (2018–2019) reported NO_*x*_/CO_2_ emission ratios for road transport (pre-ULEZ 2.6 × 10^–3^, post-ULEZ 2.2 × 10^–3^) in good agreement with those in the LAEI (2019, 2.6 × 10^–3^).^42^ LAEI emission ratios for 2016 (4.0 × 10^–3^) also agree well with those estimated from flux measurements made in Innsbruck during the same year (4.2 × 10^–3^); which at the time was a European city with a similar fleet fuel-type composition as London.^38^ The fleet turnover to cleaner vehicles, both natural and as a result of the ULEZ expansion in 2021, and reduced congestion post-COVID-19, will have reduced the emission ratio further. As such, we conservatively assume that on average, the footprint NO_*x*_/CO_2_ emission ratios for road transport are equal that in the 2025 LAEI projection (1.5 × 10^–3^).

Domestic combustion emission factors (0.25 × 10^–3^) have received some real-world verification in London and boiler age is considered in their calculation.^9,43^ They also agree well with those estimated by Karl et al. (0.2 × 10^–3^).^38^ On the other hand, nondomestic combustion has received little attention and has no real-world verification. Emissions factors used in the construction of the U.K.’s emissions inventories are taken from European EMEP/EEA guidance based in turn on somewhat outdated reference materials (Italian Ministry for the Environment, 2005).^12^ In the LAEI, an emission ratio of 1.3 × 10^–3^ is given for NO_*x*_/CO_2_. However, this does not account for recent legislation in the U.K. which limits NO_*x*_ emissions from nondomestic boilers. The Ecodesign Directive (No 813/2013) limited all new natural gas boilers ≤400 kW to a NO_*x*_ emission level of 56 mg kW^–1^ (an emission ratio of ∼0.28 × 10^–3^) from September 2018.^44^ Similarly, the 2018 Medium Combustion Plant (MCP) Directive limited plants ≥1 MW and ≤50 MW to 100 mg NM^–3^ of gas (an emission ratio of ∼0.049 × 10^–3^).^45^ Although the legislation controlling NO_*x*_ emissions from boilers became legally binding in 2018, emissions were likely achieved soon after the limits were originally announced in 2013. As such, we assume that all new boilers installed since 2014 met these new limits.

**Figure 2:**
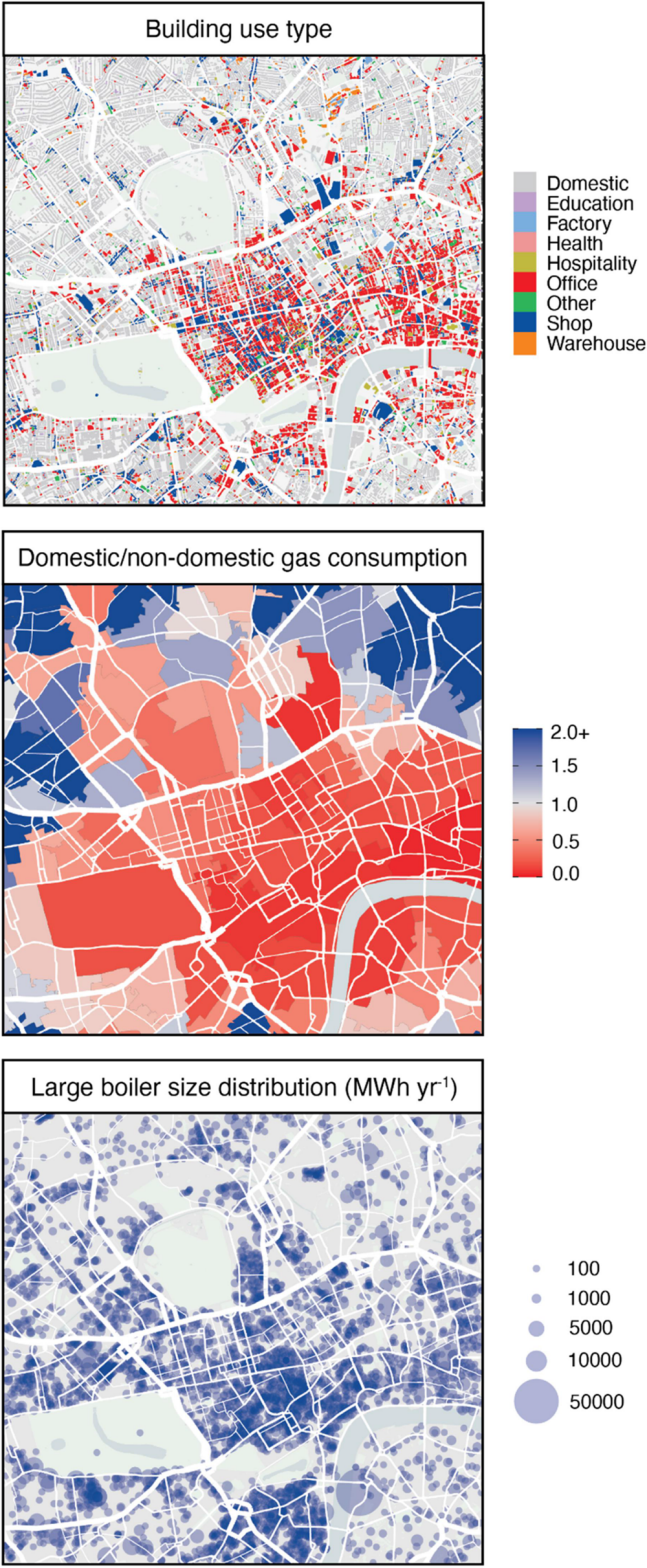
Maps of building use type, sector gas consumption and large boiler size distribution for the measurement footprint area. Map size is identical to that presented in Figure 1. Data sources are 3DStock, U.K. Department for Energy Security and Net Zero and the Decentralised Energy Master Planning program as discussed and referenced in the main text and SI. Map data courtesy of OpenStreetMap contributors, distributed under the Open Data Commons Open Database License v1.0.

The distribution of large boilers surrounding the BT Tower is shown in Figure 2. Fractions of total nondomestic heating (number of boilers multiplied by their power) for each boiler size group referred to in the legislation were extracted using the footprint model. The majority (48%) are covered by the Ecodesign Directive, with a smaller number by the MCP Directive (26%) and no Directive (26%). We use this distribution, the new Directive limits and an estimated nondomestic boiler lifetime of 15 years^46^ (or a fleet turnover of 53% since 2014) to estimate an updated nondomestic combustion NO_*x*_/CO_2_ emission ratio of 0.88 × 10^–3^, or an inventory overestimation of 51%.

#### 2.4.2 Temporal Disaggregation

Annual emission ratios were disaggregated to hour of day (*i*) for comparison to measured data. Road transport emission ratios vary diurnally due to the influence of congestion and the activity of different vehicle types. Hour of day variation in the road transport emission ratio (*R*_*t*,*i*_) was introduced using emission ratio diurnal profiles at 22 traffic monitoring sites within the ULEZ during Breathe London (2018–2019).^47^ Diurnal profiles were normalized and scaled such that the mean ratio of 1.5 × 10^–3^ was achieved. Heating emission ratios vary diurnally due to the differing activity profiles of the domestic and nondomestic sectors. An overall heating emission ratio (*R*_*h*,*i*_) was calculated for each hour of the day through eq 2
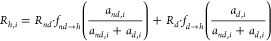
2where *R*_*nd*_ and *R*_*d*_ are the emission ratios of the nondomestic and domestic sectors, *f*_*nd*→*h*_ and *f*_*d*→*h*_ are the fractional contributions of the nondomestic and domestic sectors to gas consumption, and *a*_*nd*, *i*_ and *a*_*d*, *i*_ are normalized hourly activity factors for nondomestic and domestic gas combustion. *f*_*d*→*h*_ and *f*_*nd*→*h*_ were estimated from Middle Layer Super Output Area natural gas consumption data for the domestic and nondomestic sectors (displayed in Figure 2).^48^ The U.K. Department for Business, Energy and Industrial Strategy estimates domestic and nondomestic usage based on the size of the meter value. Although this leads to some larger domestic boilers being classified as nondomestic, and vice versa, a distinction by size is in fact preferable since this is what typically causes NO_*x*_/CO_2_ emission ratios to vary. Footprint-weighted domestic and nondomestic gas activity contributions were calculated as 34 and 66%, respectively. *a*_*d*, *i*_ was taken from the EDGAR database for the U.K.^49^*a*_nd,*i*_ was more challenging due to the lack of data available on nondomestic, in particular commercial, heating systems. The 3DStock building model was used to quantify the dominant building types within the measurement footprint. 3DStock is described in detail in the SI and provides building use classification for floor space within each unique property reference number as presented in Figure 2.^50^Table S2 presents the commercial building use activity distribution by floor space, as weighted by the measurement footprint. Offices dominate commercial buildings within the footprint at 62%, with additional contributions by unclassified commercial buildings and shops at 18 and 14% respectively. As such, heat activity data was taken from several heat meters within office buildings at University College London which is a key site in the measurement footprint. This was taken as representative of nondomestic activity and agreed well with some energy modeling studies.^51^

#### 2.4.3 Quantification

By assuming negligible contribution from other sectors (<10%), the contribution of the heating sector to total NO_*x*_ emissions (*f*_*h*→tot_) was calculated using the measured, hourly emission ratio (*R*_*m*,*i*_) in eq 3. This was done simultaneously for each hour of the diurnal and then weighted by the proportion of NO_*x*_ emissions that occurred during that hour (*a*_NO_*x*_, *i*_). A final sum across the diurnal gave the overall contribution.
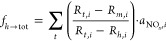
3

### 2.5 Damage Cost Estimation

Sector specific damage cost estimates in £/t of pollutant emitted were obtained from U.K. Government modeling studies as described in detail in the literature.^52^ In brief, the sensitivity of population-weighted pollutant concentrations to emissions changes are calculated with dispersion modeling and combined with concentration response functions of the cost from the resulting health and environmental impacts. Three levels of cost are estimated (low, central and high) to account for uncertainties in the health impact pathways and the differences in the valuation of a life year. We follow the appraisal guidance provided to estimate the health and environmental cost savings of decarbonisation strategies.

Total U.K. NO_*x*_ emissions from the domestic (referred to as residential in the NAEI, sector 1A4bi) and nondomestic heating sectors (sector 1A4ai) are considered and combined with different decarbonisation pathways outlined by the U.K. Climate Change Committee.^53^ We examine three potential scenarios (balanced, headwind and tailwind) and estimate the additional damage cost of NO_*x*_ produced from low-carbon hydrogen combustion in comparison to electrification and heat pumps. For each sector, U.K. wide emissions are corrected such that the boiler fleet achieves current Ecodesign Directive emissions standards. The emissions are then scaled by projected fuel use statistics in that sector assuming that hydrogen fuel and natural gas combustion have the same emission factor,^54^ and there are no further changes in application emissions standards. The cost of NO_*x*_ production from hydrogen combustion then corresponds to the sum of the emissions associated with the source for each sector combined with the sector specific damage cost for each of the sensitivity levels. The damage costs used for domestic NO_*x*_ are £12881 (£2073–£49893) and commercial NO_*x*_ are £16583 (£2469–£65232). These represent U.K. average costs for the direct combination with total U.K. emissions. However, these costs will be weighted toward high population centers of which London is the largest.

## 3 Results and Discussion

### 3.1 Temporal Trends

**Figure 3:**
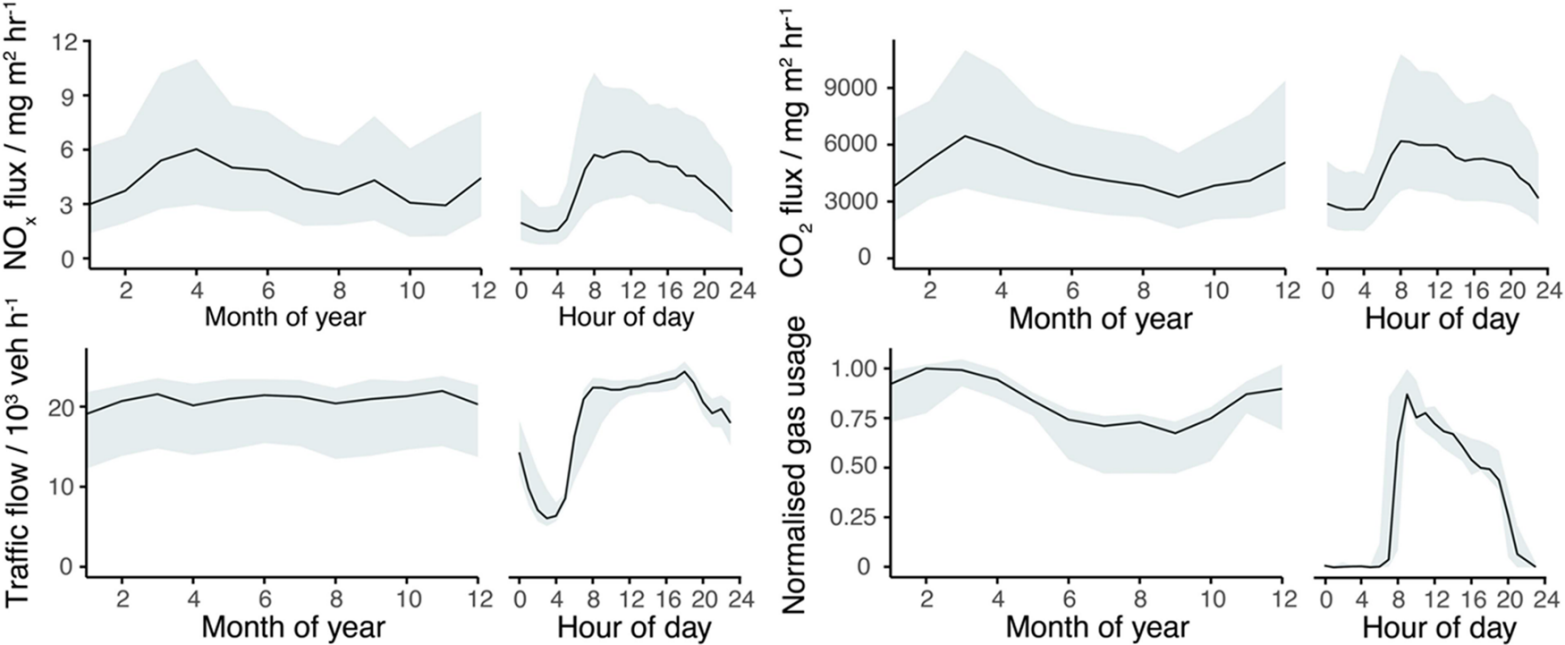
Median average monthly and diel profiles for NO_*x*_ flux, CO_2_ flux, traffic flow and natural gas usage (2021–2023). The gas usage monthly profile uses data from the North Thames LDZ which includes central London consumption. Since gas usage data has a maximum resolution of 24 h, a diel consumption profile was estimated from activity factors as discussed in the text. These are both normalized for presentation on the same axis. Shaded regions span the interquartile range (IQR) of the averaging.

Monthly and diel trends for NO_*x*_ and CO_2_ flux are presented in Figure 3 along with the road transport (traffic flow, description in SI) and energy consumption (natural gas combustion, description in SI) data for the measurement footprint area in central London. Monthly variability of NO_*x*_ and CO_2_ flux is similar and generally tracks natural gas usage, as well as the inverse of degree heating days (Figure S7). Decreasing values are seen from Spring to Autumn where ambient temperature leads to reduced combustion for building heating during warmer months. January and February have lower fluxes than expected when compared to the North Thames LDZ gas consumption. No obvious differences in meteorology (wind direction and speed, boundary layer height, and subsequent footprints) or data coverage were observed to explain the low values. However, it is noted that the North Thames LDZ is not necessarily representative of London which has its own urban microclimate. The trend could be at least partially explained by large increases in gas consumption below 285 K and a reduced proportion of days below that temperature in January and February (see Figure S8) than the surrounding months of December and March. These trends are in contrast to traffic flow which remains high throughout the year. Diel profiles for NO_*x*_ and CO_2_ now track each other closely and are representative of that of a dominant heating sector with an extended tail from the evening rush hours out-contributing the reduced heating emissions.

The observations for CO_2_ fit with current understanding; it is well established that the heat and power generation sector dominates CO_2_ emissions in major European cities. The LAEI currently attributes 80% of CO_2_ emissions within our flux footprint to the heat and power sector. In contrast, NO_*x*_ emissions in urban environments have been overwhelmingly dominated by road transport emissions for the past few decades. This has been demonstrated as recently as 2017 for central London.^15,26^

### 3.2 Source Contributions

**Figure 4:**
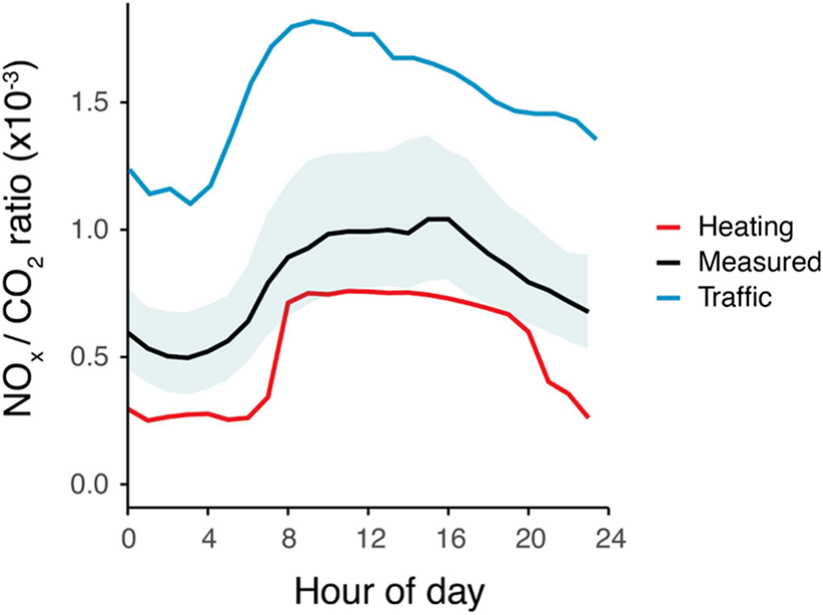
Median average NO_*x*_/CO_2_ emission ratio diel profile at the BT tower for 2021–23 in black where the shaded region represents the IQR of the averaging. Additional lines represent the traffic emission ratio (blue) and heating emission ratio (red) as discussed in the text.

The measured emission ratio during the day is shown in Figure 4 in addition to the source emission ratios. Visually, it can be explained by a dominant heating source with smaller contributions from traffic. The profile is driven by the high density of large nondomestic boilers in the measurement footprint which supply buildings (offices and shops, as opposed to health facilities and some forms of hospitality) with much-reduced gas consumption at night compared to during the working day. The small offset is a consequence of traffic emissions which contribute slightly more during evening rush hours when traffic flow is greatest.

Indeed, the calculations estimated that *f*_*h*→*tot*_ now represents 72 ± 17% of NO_*x*_ emissions. Here, the uncertainty is given as the standard error based on the monthly variation in the measured ratio. Confidence in the heating activity profiles is taken from a good agreement between gas consumption × emission factor for each sector (13% domestic, 87% nondomestic) and that calculated via the simultaneous eqs (18% domestic, 82% nondomestic). The dominance of the heating sector is further supported by the seasonal variation in the measured emission ratios. Seasonal average emission ratios of 0.87 × 10^–3^, 0.84 × 10^–3^, 0.77 × 10^–3^, 0.68 × 10^–3^ for spring, summer, autumn and winter respectively are consistent with natural gas usage driving the measured ratio. A higher proportion of gas usage in the cooler seasons results in more emissions from the heating sector and a lower measured ratio. This is as opposed to higher traffic NO_*x*_ emissions in cooler conditions which would increase the measured ratio.^55^

These observations present a major change in the dominant source from only five years previous and, as far as we know, the first observations of such in a city globally. They follow substantial reductions in road transport NO_*x*_ emissions, first calculated as 73–100% since 2017 during COVID-19 restrictions.^8^ Although these figures relate to measurements made during periods of COVID-19 restrictions, a minimal increase in flux since lockdown restrictions were lifted is suggestive that the reduction of traffic NO_*x*_ emissions may derive from some combination of the ULEZ effects, natural fleet turnover to better-performing vehicles, and a permanent change in commuting behavior post-COVID-19 in which traffic counts remain 20% lower than the baseline (see Figure S9). While every city has its own unique characteristics, London was one of the first to introduce such extensive traffic interventions. Many other European cities continue to have a high reliance on gas for heating and these observations may help them to plan accordingly should their situation be similar.

### 3.3 Emissions Inventory Comparison

**Figure 5:**
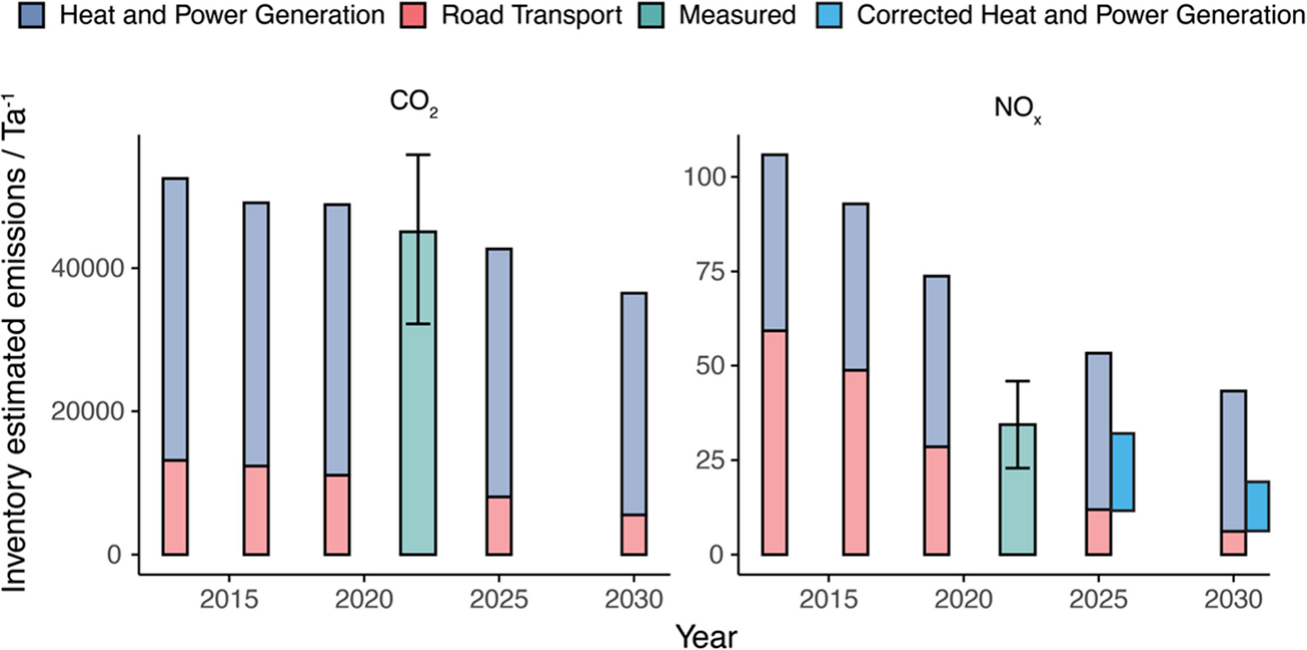
Comparison of measured and LAEI estimated annual emissions for NO_*x*_ and CO_2_ for the measurement footprint. The LAEI emissions are split by sector and the measured data is for 2022 only. Also shown is a corrected LAEI for 2025 and 2030 using heat and power generation emission factors recalculated in this study.

Section 2.4.1 calculated that NO_*x*_ emissions for nondomestic combustion are overestimated by 51% in the LAEI emissions inventory due to outdated emission factors. This is further verified when comparing measured vs inventory bulk annual emissions. Figure 5 compares emissions for 2022 vs the different LAEI years. Here, measured values are calculated from the diurnal sum of the median hour of day measured flux multiplied by the number of days in a year, to account for periods of data loss. While top-down flux estimated CO_2_ agrees well with the inventory bottom-up estimate, there appears to be an overestimation in the inventory NO_*x*_ emissions. The projected 2025 LAEI attributes an even larger proportion (78%) of NO_*x*_ to the heating sector than seen in these measurements.^9^ If the updated nondomestic emission ratio proposed here is used, a much better agreement between the measured NO_*x*_ emissions and the 2025 LAEI is seen in both magnitude (35 vs 36 kT) and heating fraction (72 vs 70%).

The wider national impact of the apparent inventory NO_*x*_ overestimate from commercial buildings is, however, likely small. Commercial combustion is a relatively minor source when considering the whole of the U.K. (around 3% of total U.K. NO_*x*_ emissions), where traffic still dominates.^56^

Since NO_*x*_ from commercial heating will be lower than is currently reported, this may help modestly in supporting the U.K. in achieving national emissions ceilings set under the international CLRTAP agreement. However, in city centers where commercial combustion becomes a more important source, these effects are more significant, particularly when inventories are used to support the modeling of future NO_2_ concentrations. Nevertheless, concentration modeling typically uses background concentration measurement sites to calibrate the dispersion model,^57^ so inventory inaccuracies may already to a degree be accounted for. Since the LAEI/NAEI follow EMEP/EEA guidance, this will likely impact most European inventories that report nondomestic heating NO_*x*_.

### 3.4 Look into the Future

Despite the large reductions in road transport NO_*x*_ emissions, all air quality sites in central London still exceed the 2021 WHO Air Quality Guidelines for NO_2_ in 2022, although most do now meet national limit values which are higher. The U.K. Government has committed to overall Net-Zero greenhouse gas emissions by 2050. This has profound long-term implications for the road transport and the heating sectors in which both will transition away from fossil fuel combustion as their primary energy source. The U.K. Climate Change Committee has a number of projections for how each sector may be decarbonized but each scenario does not necessarily achieve the same air quality benefits.^53^ Taking the heating sector as an example, overall reductions in heat demand is expected due to improved energy efficiency of buildings and behavior change. This reduction in activity would give similar reductions in emissions of GHGs and air pollutants. Similarly, the replacement of natural gas with technologies such as heat pumps would see the complete local elimination of both GHG and pollutant emissions. On the other hand, should low-carbon fuel combustion replace natural gas instead, NO_*x*_ emissions will still be present and air pollution exposure could remain an issue of concern.

**Figure 6:**
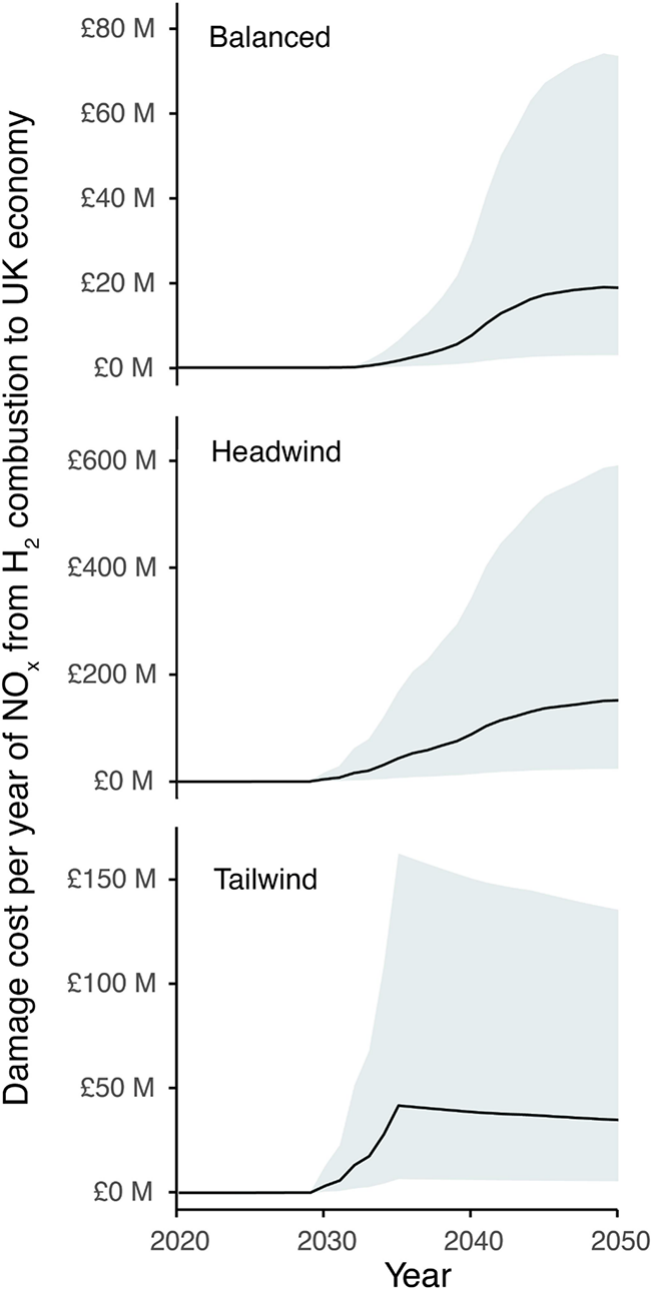
Damage cost per year of NO_*x*_ (£) from a transition containing hydrogen combustion in three different decarbonisation pathways for the U.K. heating sector vs a transition with complete electrification for heating. The black line represents the central estimate, with the shaded region following the low and high values in the sensitivity analysis.

Results from the damage cost calculations estimate a saving of £937 M (£145 M–£3.7B) per year by 2050 for zero NO_*x*_ emissions in the heating sector for the U.K. However, the use instead of low-carbon fuel boilers in each decarbonisation scenario (see Figure 6) results in a savings loss of £19 M (£3M–£75M), £35 M (£6M–£136M) and £150 M (£24M–£582M) per year by 2050 for the balanced, tailwind and headwind scenarios, respectively. These calculations isolate the impact of NO_*x*_ specifically. However, the removal of natural gas would also have an influence on primary PM_2.5_ emissions. Should a non-negligible proportion be replaced by low-carbon sources like biomass (rather than hydrogen), there will be additional major lost-savings. Nevertheless, the magnitude of the values not only highlight heating as a sector of priority when it comes to public health and air pollutant exposure, but the importance of the decisions made around the decarbonisation strategy which can play a major role in optimizing health benefits.

Specific legislation for decarbonisation of the U.K. heating sector does not yet exist, although it seems likely that installations of natural gas boilers in new homes will be banned in the near future. Decisions around the use of low carbon combustion fuels such as hydrogen and biogas have yet to be made. When considering air quality outcomes, electrification clearly supports lower urban NO_2_ and PM_2.5_ concentrations than a transition to boilers burning low carbon fuels. Additionally, there is already precedent for legislation; in California and the San Francisco Bay Area all new boilers must have zero NO_*x*_ emissions from 2027 with an estimated damage cost savings of up to $530 M per year due to NO_*x*_.^58^ An alternative approach may be a requirement for very low NO_*x*_ emissions enabled through adoption of emissions control technologies such as selective catalytic reduction in combination with low carbon fuels. However, with recent studies suggesting a supralinear relationship between pollution exposure and health,^11^ and warming climate conditions that favor more efficient secondary pollutant formation, the electrification pathways that achieve the lowest levels of emissions appear most desirable.

## Supporting Information Available

Additional details of data sets utilized in the main manuscript and features two additional tables and ten additional figures (PDF)
