# Supplementary material for: Evidence of Heating-Dominated Urban NOx Emissions
Source: Environ Sci Technol. 2025 Feb 28;59(9):4399–408. doi: 10.1021/acs.est.4c13276 (PMC11912318; doi:10.1021/acs.est.4c13276)
Supplement: Supplementary file 1 — es4c13276_si_001.pdf [file es4c13276_si_001.pdf]

Supporting information for:

Evidence of heating-dominated urban NO_x emissions

Samuel J. Cliff [a,h,*], Will Drysdale[a,b], Alastair C. Lewis[a,b], Sarah J. Moller[a,b], Carole Helfter[c], Stefan Metzger[d,e], Rob Liddard[f], Eiko Nemitz[c], Janet F. Barlow[g], and James D. Lee[a,b,*]

[a] Wolfson Atmospheric Chemistry Laboratories, University of York, York, YO10 5DQ, United Kingdom

[b] National Centre for Atmospheric Science, university of York, York, YO10 5DQ, United Kingdom

[c] UK Centre for Ecology and Hydrology, Bush Estate, Penicuik, EH26 0BQ, United Kingdom

[d] AtmoFacts LLC., 3570 Larkspur Court, Longmont, CO 80503, United States

[e] Department of Atmospheric and Oceanic Sciences, University of Wisconsin-Madison ,1225 W Dayton Street, Madison, WI 53711, United States

[f] UCL Energy Institute, University College London, London, WC1E 6BT, United Kingdom

[g] Department of Meteorology, University of Reading, Reading, RG6 6BB, United Kingdom

[h] Now at: Department of Civil and Environmental Engineering, University of California, Berkeley, CA 94720, USA

* Corresponding authors. Email: samcliff@berkeley.edu, james.lee@york.ac.uk

This supporting information contains 9 pages of additional details and analysis with 2 tables and 10 figures.

S1. Additional data on emissions drivers

S1.1 Traffic

Hourly traffic loads surrounding the BT Tower were calculated by summing the traffic load from each of the 24 Automatic Traffic Counters (ATCs) within the flux footprint. Data was provided by the Operational Analysis Department, Transport for London (TFL) via a freedom of information request.

S1.2 Natural gas usage

Natural gas demand for the North Thames (NT) local distribution zone (LDZ) (Demand, actual daily metered, LDZ (NT), D+1) which London resides in was collated from the National Gas Data Portal.[1] Annual domestic and non-domestic gas usage by middle super output layer (MSOA) was obtained from the Department for Business, Energy and Industrial Strategy.[2] Hourly activity data for non-domestic gas consumption was estimated from utility meters within University College London (see further discussion in the main text). This was provided by their sustainability team and data for several office buildings for the whole of 2022 was averaged and used in this study. Hourly activity data for domestic gas consumption was obtained from the EDGAR database for UK domestic buildings.[3]

S1.3 Building types

The 3DStock model was used to identify building use sectors within the BT Tower measurement footprint. 3DStock is the model upon which the London Building Stock Model is constructed and brings together several open (and a few limited) access datasets to create a disaggregate, geometrical, geospatial model of buildings and their uses in England and Wales.[4] The model is primarily for use in the modeling of energy use in building stocks and thus includes detailed information on activities and the floor areas these occupy, including where multiple

activities share a building, such as apartments above offices, above retail. The system used to classify activities, as used in this current work, is called 'CaRB2'.

S1.4 Large heat sources

Consumption data for the production of heat for buildings with large boilers in central London was collated as part of the Decentralised Energy Master planning program (DEMaP) for the Mayor of London and used in this study.[5]

Table S1: Continuous wavelet transformation parameters used in eddy4R.

Parameter	Value
δt	0.2 s
Zero padding	TRUE
δj	0.125
J_{max}	110
Mother wavelet	Morlet
COI filter	No filter
Wavelet maximum scale	60 minutes
Averaging period	1 minute
Averaging window	5 minutes

Table S2: CaRB2 classification distribution of commercial building floor space weighted by location within the footprint.

Building use category	% commercial floor space in footprint
Education	0.75
Factory	0.57
Health	1.02
Hospitality	1.84
Office	61.97
Other	0.97
Shop	14.29
Unclassified commercial	18.29
Warehouse	0.33

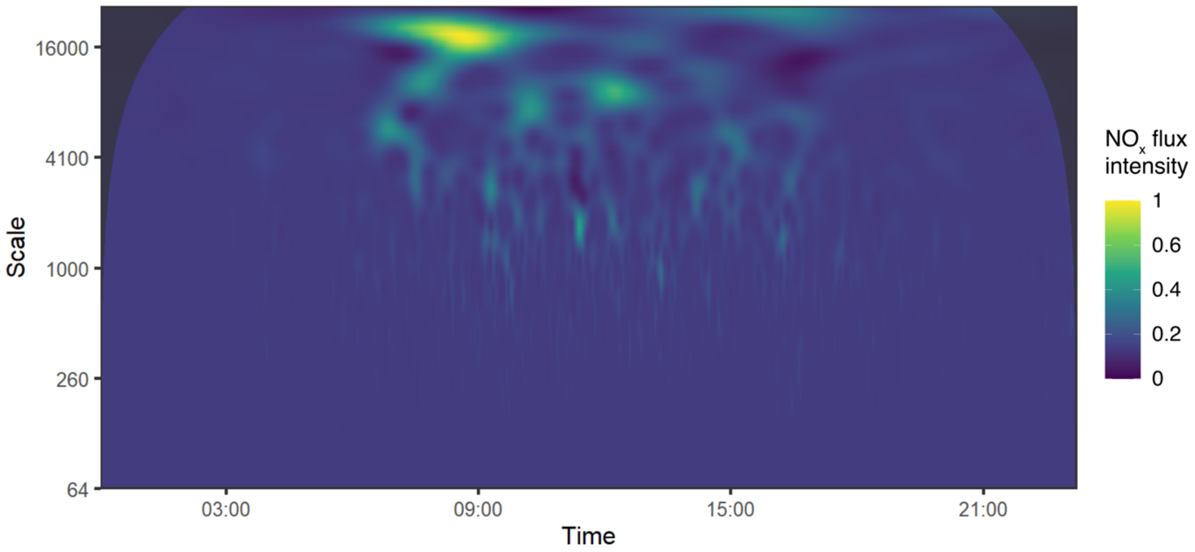

Figure S1: An example wavelet cross spectrum for 24 hours of NO_x data from the BT Tower during a typical Summer's day. Yellow colors represent areas of high emission compared to blue colors of low emission. Shaded in black is the wavelet cone of influence.

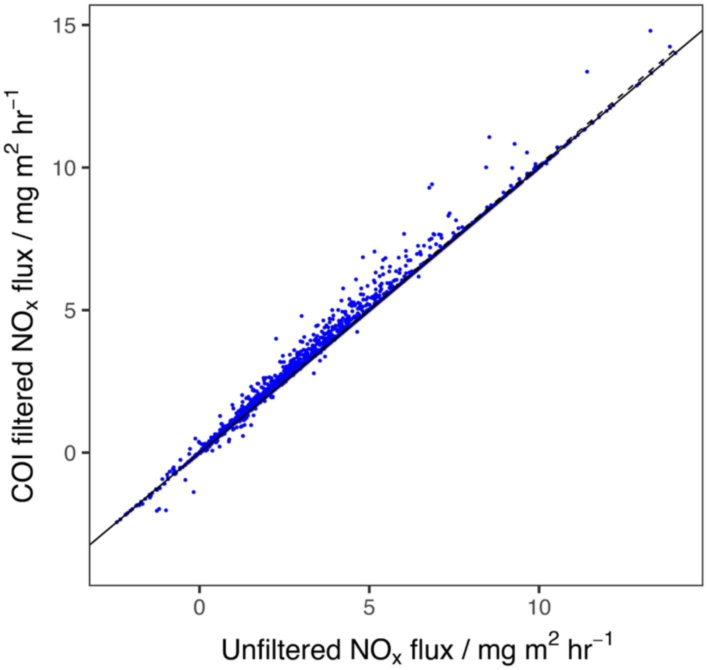

Figure S2: Regression plot of NO_x flux calculated with no cone of influence (COI) filter, and that with a filter applied in which periods with > 80% of the spectral power within the COI are removed, for a two week measurement period.

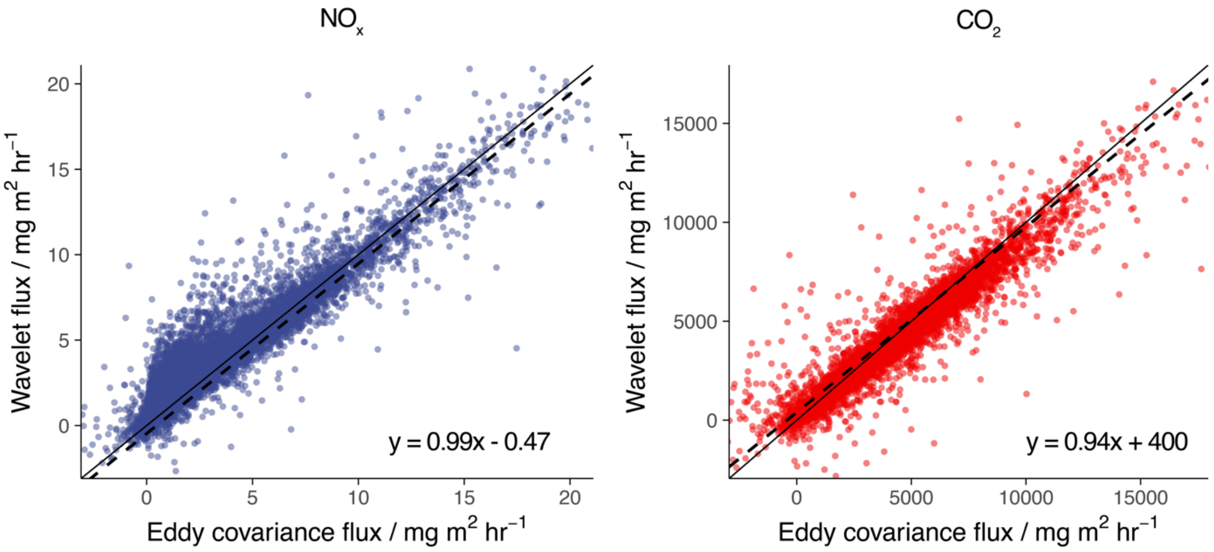

Figure S3: Correlation between NO_x and CO_2 flux calculated from continuous wavelet transformation and traditional eddy covariance.

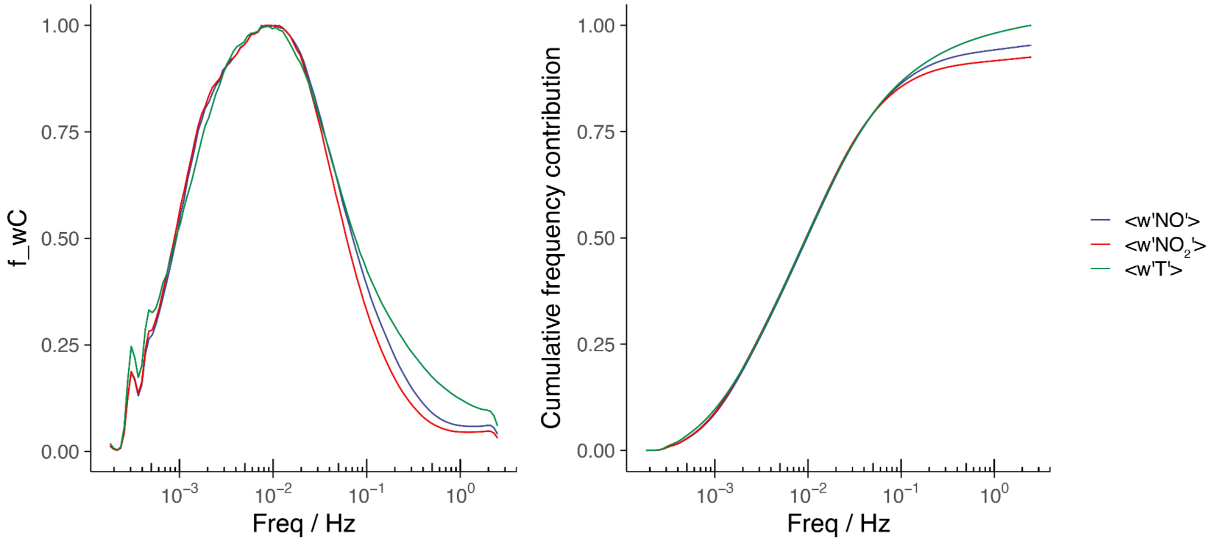

Figure S4: Campaign average co-spectra of normalized covariance between instantaneous vertical wind speed with NO, NO_2 and temperature.

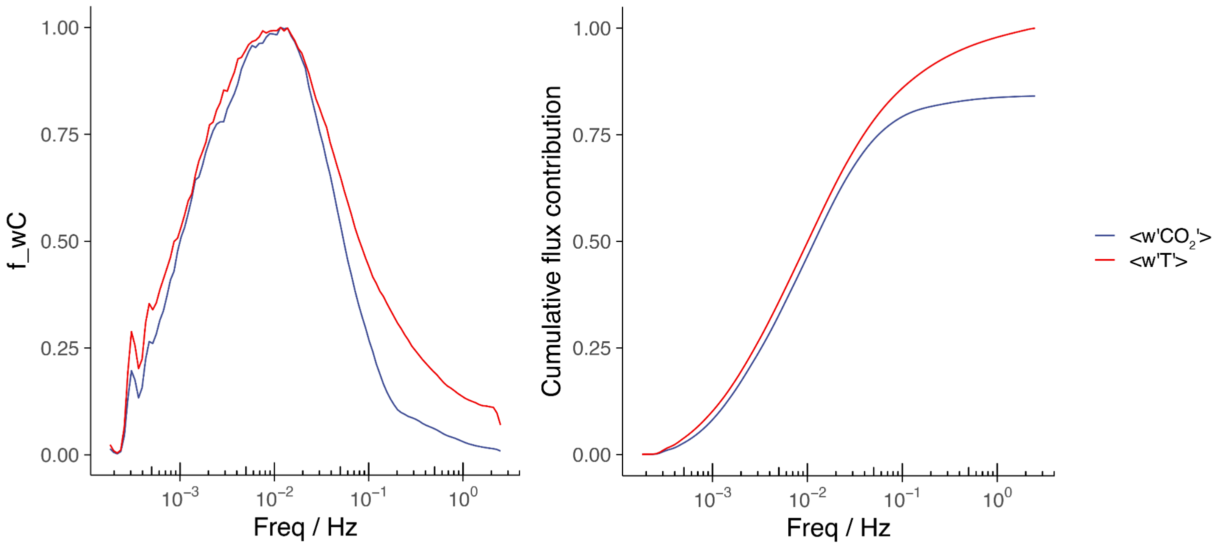

Figure S5: Campaign average co-spectra of normalized covariance between instantaneous vertical wind speed with CO_2 and temperature.

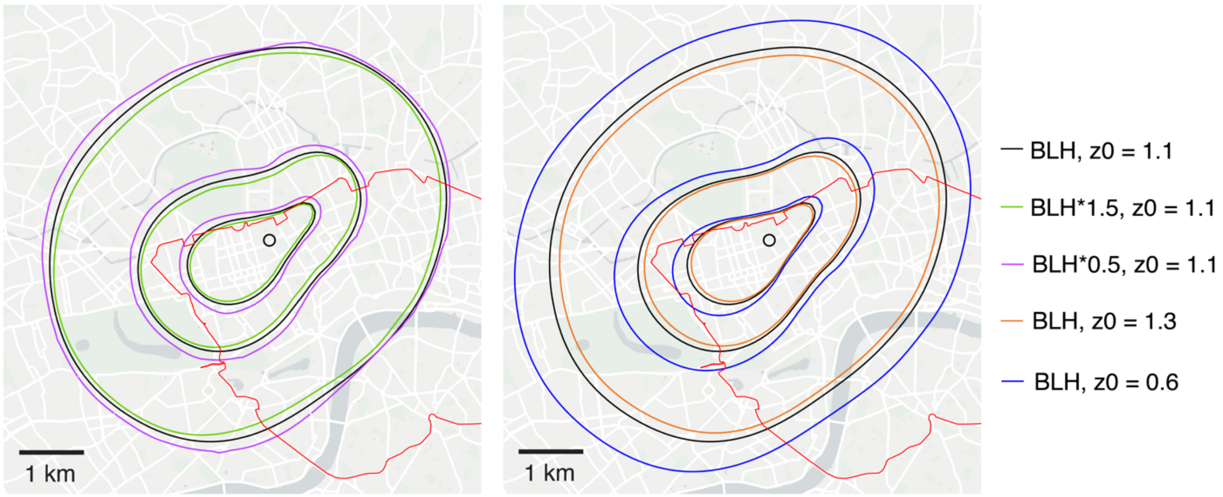

Figure S6: Footprint sensitivity tests for left: boundary layer height (BLH) multiplied by 0.5 and 1.5, and right: surface roughness length (z0) of 0.6 and 1.3 as determined from Drew et al.,[6] The base case is standard BLH and z0 of 1.1 as discussed in the main text. Map data courtesy of OpenStreetMap® contributors, distributed under the Open Data Commons Open Database License v1.0.

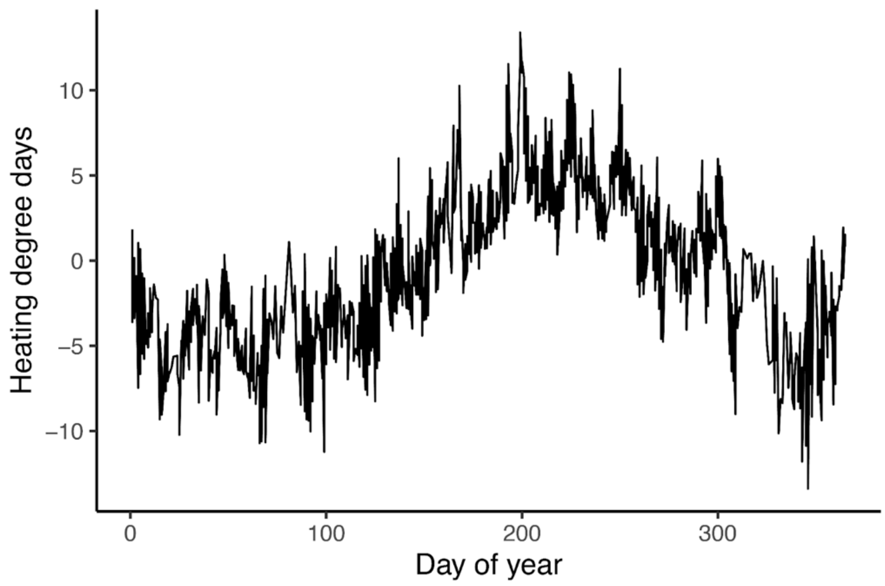

Figure S7: Heating degree days for temperatures and days during valid flux measurements at the BT Tower.

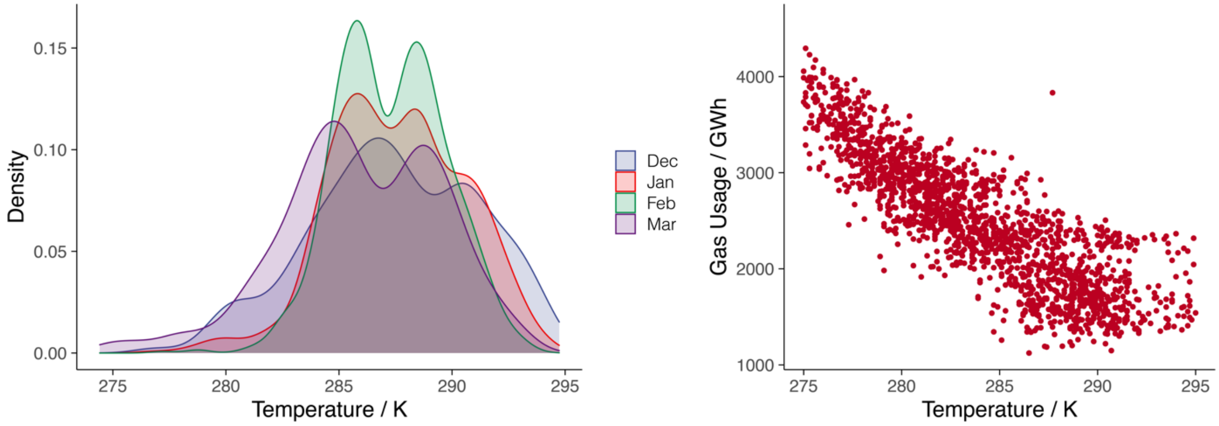

Figure S8: Distribution of temperatures experienced at the BT Tower for Dec-March with comparative UK gas combustion within that temperature range.

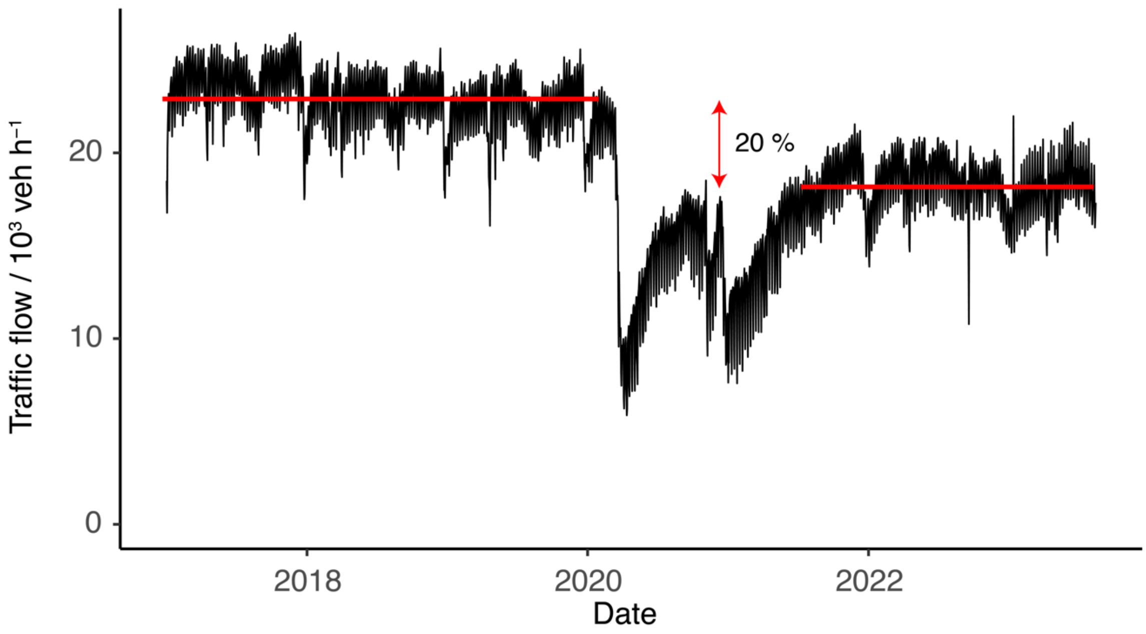

Figure S9: Daily average traffic flow time series for the 24 automatic traffic counters surrounding the BT Tower. Highlighted in red is the 20 % lower traffic flow during the measurement campaign presented in this analysis than pre-COVID19 that persisted despite lifting of lockdown restrictions.

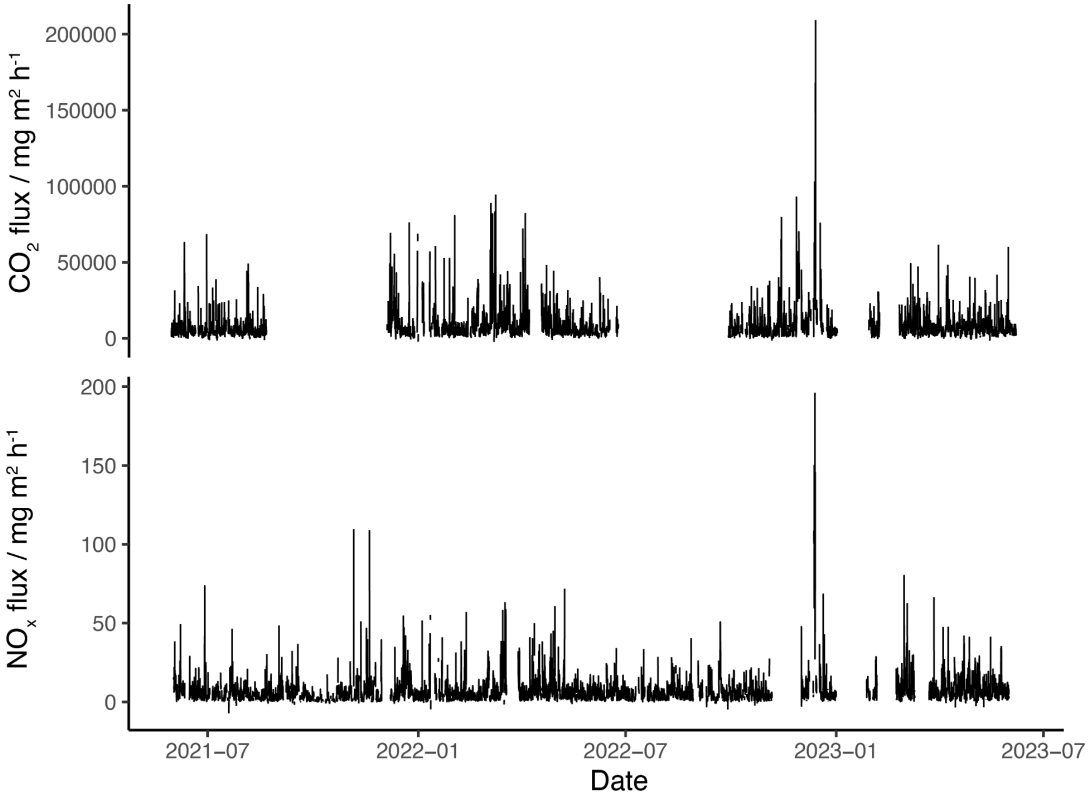

Figure S10: Time series for daily average CO_2 and NO_x flux for the analysis period in the main text.

References

(1) *National Gas Transmission Data Portal*. https://data.nationalgas.com/ (accessed 2024-11-20).
(2) Department for Energy Security and Net Zero. *Lower and Middle Super Output Areas gas consumption*. https://www.gov.uk/government/statistics/lower-and-middle-super-output-areas-gas-consumption (accessed 2024-11-20).
(3) Crippa, M., Solazzo, E., Huang, G., Guizzardi, D., Koffi, E., Muntean, M., Schieberle, C., Friedrich, R., Janssens-Maenhout, G. High Resolution Temporal Profiles in the Emissions Database for Global Atmospheric Research. *Sci. Data* **2020**, *7* (1), 121.
(4) Steadman, P., Evans, S., Liddiard, R., Godoy-Shimizu, D., Ruyssevelt, P., Humphrey, D. Building Stock Energy Modelling in the UK: The 3DStock Method and the London Building Stock Model. *Build. Cities* **2020**, *1* (1).
(5) Greater London Authority. *London Heat Map - London Datastore*. https://data.london.gov.uk/dataset/london-heat-map (accessed 2024-11-20).
(6) Drew, D. R., Barlow, J. F., Lane, S. E. Observations of Wind Speed Profiles over Greater London, UK, Using a Doppler Lidar. *J. Wind Eng. Ind. Aerodyn.* **2013**, *121*, 98–105.
